# Will the Articular Surface Collapse After the Fixation of a Tibial Plateau Fracture?

**DOI:** 10.7759/cureus.91099

**Published:** 2025-08-27

**Authors:** Mohammad Monir, Ahmed Elkohail, Mohamed Elbanna, Ali Soffar, Ahmed Al-Feeshawy

**Affiliations:** 1 Orthopaedics and Trauma, General Organization For Teaching Hospitals and Institutes, Cairo, EGY; 2 Orthopaedics and Trauma, Princess Royal University Hospital, King's College Hospital NHS Foundation Trust, London, GBR; 3 Trauma and Orthopaedic Surgery, Princess Royal University Hospital, King's College Hospital NHS Foundation Trust, London, GBR; 4 Orthopaedics and Trauma, Cairo University Hospitals, Cairo, EGY

**Keywords:** articular surface collapse, rasmussen score, secondary articular depression, tibial plateau, weight–bearing

## Abstract

Background: Tibial plateau fractures present significant challenges, especially when joint depression is accompanied by metaphyseal comminution. The primary objective in these fractures is to attain, if feasible, an anatomic reduction of the articular surface, thereby restoring joint congruity and mechanical alignment to enhance functional outcomes and decrease the incidence of post-traumatic arthritis. Fractures with significant depression of the articular surface necessitate the elevation of the depressed fragments and stable internal fixation.

Objective: This study aimed to assess prospectively postoperative articular congruity and determine the articular surface collapse following open reduction and restoration of depressed tibial plateau fractures. The analysis of results was made in terms of age of patients, sex distribution, type of fracture, and special habits. The second objective of the study was to evaluate the surgical and patient-related factors associated with this drawback. The data were analyzed to detect any statistically significant correlation between these parameters and clinical and radiographic outcomes using Rasmussen's clinical and radiological grading systems in a six-month follow-up.

Methods: Patients with tibial plateau fractures were selected based on inclusion and exclusion criteria, and these fractures were categorized according to the Schatzker classification based on post-traumatic anteroposterior and lateral X-ray films. In addition to that, all patients had preoperative CT scans of the injured knee. The sample size consisted of 40 cases with a history of proximal leg or knee trauma. All cases underwent open reduction and restoration of the articular surface. Depression of the joint surface was elevated under visual control; a buttress plate and screws were used for internal fixation. For inclusion in the present analysis, two conditions had to be satisfied: adequate reduction with plate fixation and a follow-up period of at least six months. Radiological follow-up included an early postoperative X-ray and CT scan, serial follow-up X-rays, and an X-ray and CT scan at six months.

Results: Fourteen (35%) patients assessed experienced postoperative articular surface collapse during the follow-up evaluation. A statistically significant association was found between postoperative articular surface collapse and the following variables: age, preoperative articular surface fragmentation, use of bone graft, smoking, and early weight-bearing. Collapse correlated strongly with clinical outcomes, including significant knee pain (p<0.001) and reduced walking capacity. The presence of collapse also adversely affected the radiographic scores at follow-up (p < 0.001).

## Introduction

Tibial plateau fractures (TPFs) encompass the articular and meta-epiphyseal proximal tibia. The considerable displacements among bone fragments, the resulting impaction and depression of the subchondral cancellous bone (SCB), and the unavoidable injury to articular cartilage make their care difficult [1].

TPFs take place with a frequency of 10.3 in 100,000 yearly and account for approximately 1% of all fractures [2]. These fractures are infrequent, exhibiting a bimodal distribution in both genders. The prevalent injury mode constitutes pedestrians being injured by motorized vehicles (30%), followed by low-energy falls (LEFs), comprising 22% of TPFs. In elderly individuals, they typically occur due to unintentional LEFs, particularly in the female population, with lateral tibial plateau fractures occurring more frequently than those of medial or bicondylar injuries. In the younger demographic, high-energy injuries are predominant. Young male individuals exhibit greater vulnerability, indicating a reversal of gender predominance. Motor vehicle collisions, athletic activities, and falls from heights are prominent modes of trauma [3].

The trabecular bone architecture at the proximal end of the tibia can enhance the comprehension of various fracture types and their prevalence. According to Dupare and Ficat [4] and Roberts [5], the cancellous bone at the upper tibia has a trabecular arrangement depicted in coronal sections as follows: Vertical trabeculae run from the medial metaphyseal cortex toward the medial plateau, and intersecting transverse trabeculae are located beneath the articular surface. The longitudinal trabeculae have reduced density and extend from the lateral metaphyseal cortex toward the lateral plateau. The transverse trabeculae, located just beneath the articular surface, are also slender. This notable distinction elucidates the corresponding susceptibility of the lateral plateau to injuries.

Approximately 60% of the axial load is borne by the medial side, resulting in denser SCB relative to the lateral. Accordingly, medial plateau fractures are typically attributed to high-energy trauma and are often associated with collateral soft-tissue injuries, including lateral ligamentous complex disruption, peroneal nerve compromise, or vascular insult involving the popliteal artery. The elevation of the lateral plateau relative to the medial leads to a slight varus alignment of the tibial plateau with respect to the tibial shaft. The structural disparities, along with the anatomical valgus knee axis and the predominance of lateral vector forces during trauma, predispose the lateral fractures [3].

Trauma leading to such injuries may arise from medially applied forces producing valgus deformities (commonly referred to as “bumper fractures”) or from lateral forces causing varus deviation, direct axial compression, or a combination thereof. The corresponding femoral condyle imparts both shear and compressive forces on the impacted tibial surface, culminating in split, depressed, or combined fracture patterns [6].

The fracture pattern generated is contingent upon both force intensity and the flexion angle of the knee during impact. Research indicates that the application of abduction force results in a split or cleavage fracture pattern. Exerting compression forces leads to the formation of compression fractures, which in turn cause a depressed region and articular injury that resembles a mosaic pattern [7].

Younger people are more likely to have pure split fractures since the SCB withstands compressive femoral loading; however, the shear component induces split-type fractures. With aging and the onset of osteopenia, cancellous bone loses its compressive integrity, giving rise to combined split-depression fractures, especially in patients over 50. These are often triggered by low-energy mechanisms such as slip-and-fall incidents [8].

A prototypical example of this is the lateral-sided “bumper” injury, typically initiated by a lateral force inducing valgus stress and causing the femoral condyle to drive into the lateral condyle. In high-energy scenarios, the magnitude of force may fragment the surface into multiple segments. Biomechanical studies have shown that axial force over 8000 pounds resulted in severely comminuted fractures. This mechanism is frequently observed following a fall from height or a motor vehicle collision with an axial stress applied to an extended knee [9].

Conservative treatment is applicable only to uncomplicated, undisplaced fractures, comprising only a minority of cases, or to patients with low functional demands and significant comorbidities [10]. The substantial appositional cancellous bone along the cross-sectional area and robust vascularization of the proximal tibia render a nonunion TPF unlikely [11].

The prevailing agreement for young patients with this type of injury is to pursue surgical intervention (open reduction and internal fixation (ORIF)), focusing on anatomical fracture reduction, rigid internal fixation, and early mobilization. The surgical indications and objectives are broadening to be more applicable to patients over 55 years, demonstrating positive outcomes even in the setting of osteopenia/osteoporosis, concurrent medical conditions, or pre-existing degenerative joint conditions [4]. Knee instability, malalignment of the varus or valgus more than 10°, articular step-off of 2-10 mm, condylar widening greater than 5 mm, or related soft tissue damage are generally recognized indications for surgery [12].

ORIF using plates and screws is regarded as the gold standard treatment method. Some authors recommend arthroscopic-assisted reduction combined with internal fixation in particular instances, specifically pure depressed fractures. However, concerns about the potential development of compartment syndrome due to extravasation of irrigation fluid into the leg compartments, as well as increased surgical time and logistical challenges, have constrained the widespread adoption of this technique [13]. Key factors influencing the prediction of both immediate and long-term outcomes include age, existing health issues, smoking history, occupation, functional capacity, and personal expectations [1].

Aim of the study

The study's purpose was to evaluate the postoperative depression of the articular surface in TBFs as a drawback following open reduction, reconstruction of the articular surface, and internal fixation using a buttress plate and screws, with or without bone grafting. The objective is to delineate the predominant etiologies contributing to this postoperative issue and assess its implications on functional and radiological outcomes. Outcome assessment was performed utilizing the modified Rasmussen scoring system, encompassing both clinical and imaging parameters.

## Materials and methods

This was a prospective cohort study. All procedures were performed at the Kasr Al-Ainy University Hospitals and the Ahmed Maher Teaching Hospital in Cairo, Egypt. The study was approved by the Postgraduate Affairs and research Office Committee, Faculty of Medicine, Cairo University (ID: M.Sc-18467-2020 dated March 28, 2020). Informed written consent was obtained from all study participants.

Patients with tibial plateau fractures were prospectively selected from a hospital society based on defined inclusion and exclusion criteria, and these fractures were categorized according to the Schatzker classification based on post-traumatic anteroposterior and lateral X-ray films. In all cases, preoperative CT imaging of the affected knee was performed to enable precise assessment of fracture configuration. Eligibility for inclusion in the study required two criteria to be met: (i) confirmation of satisfactory reduction and fixation, and (ii) a minimum follow-up period of six months.

The sample size consisted of 40 cases with a history of proximal leg or knee trauma. All cases underwent open reduction and restoration of the articular surface. Articular depressions were elevated under direct visualization. In 24 cases, the resulting metaphyseal defect was filled with autologous bone harvested from the iliac crest. The remaining 16 patients either had minimal defects not necessitating filling or declined autograft harvesting. In all cases, internal fixation was achieved using a buttress plate with supplementary screws.

Patients were monitored on a weekly basis until fracture union, followed by comprehensive evaluations at three and six months postoperatively, with the six-month point representing the final follow-up. Clinical follow-up included assessment of pain levels, ambulatory capacity, knee range of motion (ROM), extension, and joint stability. Radiological evaluation involved immediate postoperative X-rays and CT scans, with additional follow-up imaging and another CT at the six-month interval.

Postoperative depression of the articular surface was assessed on serial anteroposterior and lateral radiographs. The magnitude of depression was quantified by drawing a reference line along the adjacent intact articular surface and measuring the vertical distance to the point of maximal depression. This method mirrored the reproducible radiographic technique described by Kumar and Whittle [14]. Uniform radiographic technique across cases allowed for comparative analysis [5].

In bicondylar fracture cases, a horizontal line at the level of the femoral condyles was used as a reference, with a second line drawn parallel to it through the tibial spine base. Measurements were taken to the deepest depressed point on each plateau. CT was considered the definitive modality for intra-articular evaluation, particularly when further characterization of articular involvement was required in complex or depression-type fractures.

Throughout the study, measurement of postoperative depression was conducted via both serial radiographs (anteroposterior and lateral) and CT scans obtained postoperatively and at the six-month follow-up. All imaging was assessed independently by blinded orthopedic and radiology specialists to determine interobserver reliability. Each observer measured the degree of articular depression across the 40 patients, with the collected data subjected to subsequent statistical analysis.

For this study, we established specific patient selection criteria to ensure appropriate candidates for surgical intervention and subsequent analysis. Eligible participants included skeletally mature patients aged 18 years or older who presented with closed tibial plateau fractures. We excluded several patient populations to maintain study integrity and safety considerations. Patients with concomitant injuries that could interfere with standard rehabilitation protocols, particularly those with ipsilateral femoral fractures that would compromise range of motion exercises or weight-bearing activities, were not included. Additionally, we excluded individuals with significant medical comorbidities that would contraindicate surgical intervention, as well as those presenting with associated neurovascular compromise that could confound treatment outcomes. Patients with pre-existing lower limb paralysis were deemed inappropriate candidates, as were those with open tibial plateau fractures or pathological fractures, which represent distinct clinical entities requiring alternative management approaches. Finally, skeletally immature patients and those treated with alternative fixation methods such as external fixation were excluded to maintain homogeneity in both patient demographics and surgical technique within our study cohort.

Postoperative protocol

All patients received postoperative care that included intravenous administration of antibiotics and analgesics at 12-hour intervals for a duration of three days. Surgical drain tubes were removed after 48 hours. The initial wound assessment was carried out on postoperative day three, followed by inspections every three days. Suture removal was performed on the tenth postoperative day.

Initiation of active knee mobilization and static quadriceps strengthening exercises began on the first postoperative day, tailored to the patient's pain tolerance and level of cooperation. Patients were mobilized using non-weight-bearing ambulation, assisted either by axillary crutches or a walking frame, depending on the individual's age and overall physical condition. Weight-bearing activities were strictly deferred for a minimum of ten weeks and until radiological confirmation of fracture healing was obtained.

Post-discharge follow-up assessments were scheduled at three, six, and 12 weeks, as well as at the six-month mark. Serial plain radiographs were obtained during these visits to monitor fracture healing. Knee ROM was also measured and documented at each follow-up.

Clinical and radiographic evaluations were conducted utilizing the Rasmussen Clinical Score and the Rasmussen Radiological Grading System (Tables 1, 2). The final outcomes were systematically analyzed and recorded based on the six-month follow-up data.

**Table 1:** Rasmussen's functional grading system Rasmussen’s functional grading system for tibial plateau fracture outcomes. Scoring criteria adapted from Rasmussen et al. (1973) [15].

Parameters	Score
Pain	
Non	6
Occasional	5
Stabbing pain in certain position	3
Constant pain after activity	1
Significant rest pain	-3

Walking capacity	
Normal walking for age	6
Walking outdoor (>1h)	5
Walking outdoor (15m-1h)	3
Walking outdoor (<15m)	1
Walking indoor only	0
Wheelchair	-3
Knee extension	
Normal	4
Lack of extension (<10^0^)	2
Lack of extension (>10^0^)	0
Lack of extension (<20^0^)	-2
Total range of movement	
full	6
At least 120^0^	5
At least 90^0^	4
At least 60^0^	2
>60^0^	0
Stability	
Normal at extension and 20^0^ flexion	6
Abnormal stability at 20^0^ flexion	4
Unstable in extension (<10^0^)	2
Unstable in extension (>10^0^)	0
Quadriceps power	
Grade 5	2
Grade 3-4	1
Grade <3	0
Maximum score	30
Excellent	28-30
Good	24-27
Fair	20-23
Poor	<20

**Table 2:** Rasmussen's radiological grading system Rasmussen’s radiological grading system for tibial plateau fractures [15]. Criteria include articular depression, condylar widening, and angulation, scored 0–10.

Parameters	Score
Articular depression	
Non	3
<5mm	2
5-10mm	1
>10mm	0
Condylar widening	
None	3
<5mm	2
5-10mm	1
>10mm	0
Valgus/Varus angulation	
None	3
<10^0^	2
10-20^0^	1
>20^0^	0
Osteoarthritis	
None / No progression	1
Progression to grade 1	0
Progression > grade 1	-1
Maximum score	
Excellent	9-10
Good	7-8
Fair	5-6
Poor	<5

Statistical analysis

Data were coded and analyzed using the IBM SPSS Statistics for Windows, version 28 (IBM Corp., Armonk, NY, USA). Descriptive statistics, including mean and standard deviation, were used to summarize quantitative variables, while categorical variables were summarized using frequencies and percentages. Comparisons between groups were performed using the unpaired t-test [16]. For categorical data, the Chi-square (χ²) test was applied. When the expected frequency in any category was less than five, Fisher's exact test was utilized. To identify independent predictors of articular surface collapse, logistic regression analysis was conducted [17]. A p-value of less than 0.05 was considered statistically significant.

## Results

A total of 40 patients were included in the study. The average age of the examined patients was 50.85 years with a standard deviation of 10.05 years. Concerning sex, over half the participants were male (52.5%). The majority of participants were non-smokers, accounting for 30 patients (75%), while 10 patients (25%) reported active cigarette smoking at the time of enrollment. This distribution reflects a predominantly non-smoking patient population, which may have implications for wound healing and postoperative recovery outcomes in the context of tibial plateau fracture management (Table 3).

**Table 3:** Demographic characteristics of included patients (N=40)

Characteristics	Values
Age (years), mean ±SD (range)	50.85 ±10.05 (29.00-68.00)
Sex, n (%)	
Male	21 (52.5%)
Female	19 (47.5%)
Smoking, n (%)	
Non-smokers	30 (75%)
Active smokers	10 (25%)

Regarding the mode of trauma, the most frequent was road traffic accidents (n=26, 65.0%), followed by a slide and fall (n=14, 35.0%). Fracture classification according to the Schatzker system revealed distinct patterns within our patient cohort. The most prevalent fracture type was Schatzker Type II, which was observed in 26 patients, representing approximately two-thirds of the study population (65%). Schatzker Type III fractures were identified in 12 patients, accounting for nearly one-third of cases (30%). The remaining two patients presented with Schatzker Type V fractures, comprising the smallest subset at 5% of the total cohort.

Patients were categorized preoperatively according to their fracture configuration as simple or complex comminuted fractures in relation to the degree of depression, separation, and fragmentation. Simple fracture patterns predominated, occurring in 27 patients and representing more than two-thirds of all cases (67.5%). In contrast, complex or comminuted fracture configurations were documented in 13 patients, accounting for approximately one-third of the cohort (32.5%).

During the surgical treatment of patients, an autogenous bone graft was used in most of the participants (n=24, 60%). The remaining 16 patients underwent surgical fixation without the use of bone grafting materials, accounting for 40% of cases. The joint surface was reconstructed and reduced without a graft in response to the patient’s refusal or due to the decision taken in the departmental meeting for simple fractures with joint surface depression not exceeding 4 mm; the joint surface was elevated and anatomically reduced through a cortical window and without grafting. 

Weight bearing was not permitted until 10 weeks and until definitive radiological signs of fracture union. However, bearing weight started early in some patients who rushed to walk, violating the instructions. The majority of patients (n=30, 75%), were restricted from weight-bearing activities for more than 10 weeks following surgical intervention. This extended non-weight-bearing period typically reflected the presence of complex fracture patterns requiring prolonged protection to ensure adequate bone healing and maintenance of articular reduction. A smaller subset of patients (n=5, 12.5%) was able to commence earlier mobilization protocols, initiating partial weight-bearing activities between six to eight weeks postoperatively. An additional five patients (12.5%) progressed to full weight-bearing status within the same six-to-eight-week timeframe.

Total radiological score at six months

At the six-month postoperative evaluation, radiological outcomes were assessed using Rasmussen's grading system, which demonstrated predominantly favorable results across the patient cohort. The majority of patients achieved excellent radiological outcomes, with 23 individuals (57.5%) meeting the highest grading criteria, indicating optimal fracture healing, maintenance of articular congruity, and absence of significant complications. Good radiological results were observed in nine patients (22.5%), representing satisfactory healing with minor residual changes that did not significantly compromise joint function. Fair outcomes were documented in eight patients (20%), suggesting acceptable healing despite some persistent radiological abnormalities such as mild articular step-offs or joint space narrowing. Notably, no patients in this series were classified as having poor radiological outcomes at the six-month follow-up period. These results indicate that the surgical management approach employed in this study achieved satisfactory radiological healing in the vast majority of cases, with nearly 80% of patients demonstrating either excellent or good outcomes according to established radiological criteria.

Total functional score at six months

Functional outcomes at six months post-surgery were evaluated using Rasmussen's functional scoring system, revealing generally positive results across the patient population. More than half of the patients achieved excellent functional outcomes, with 21 individuals (52.5%) demonstrating optimal knee function with minimal to no limitations in daily activities and returning to pre-injury functional levels. Good functional results were documented in 10 patients (25%), indicating satisfactory knee function with only minor restrictions that did not significantly impact quality of life or routine activities. Fair functional outcomes were observed in six patients (15%), representing acceptable function despite some persistent limitations such as mild pain, stiffness, or reduced range of motion that occasionally interfered with demanding activities. A small subset of three patients (7.5%) experienced poor functional outcomes, characterized by significant functional impairment, persistent pain, or substantial limitations in activities of daily living.

Postoperative articular surface depression

During the follow-up evaluation, radiological assessment revealed varying degrees of articular surface depression among the patient cohort. The majority of patients, comprising 26 individuals (65%), demonstrated no evidence of articular surface collapse, indicating successful maintenance of joint congruity following surgical intervention. However, articular surface depression was identified in 14 patients (35%). Among those with measurable depression, eight patients (20%) exhibited mild articular surface collapse measuring less than 5 mm, which typically represents a clinically acceptable reduction with minimal functional impact. A smaller subset of six patients (15%) demonstrated more significant articular depression, ranging from 6-10 mm, indicating substantial loss of joint surface integrity that could potentially compromise long-term joint function and predispose to post-traumatic arthritis.

Relations with articular surface collapse

Based on the results of the studied patients. Preoperative fracture displacement-comminution, smoking habit, use of bone grafting, and weight-bearing time demonstrated a significant correlation with the articular surface, as indicated by a significant P value (Table 4). There was significant associations with absence of bone grafting (71.4% vs 23.1% with grafting, p=0.003), complex preoperative fracture patterns (71.4% vs 11.5% simple fractures, p<0.001), smoking history (50% vs 11.5% non-smokers, p=0.018), and premature weight-bearing (35.7% initiating full weight-bearing before 10 weeks vs 0% compliant patients, p<0.001).

**Table 4:** Studied factors related with articular surface collapse. Statistical analysis was performed using chi-square or Fisher's exact tests with significance at p<0.05 RTA: road traffic accident

Parameters		Articular surface collapse				
		Yes		No		P value
		Freuqency	Percentage	Frequency	Percentage	
Gender	Male	6	42.9%	15	57.7%	0.370
	Female	8	57.1%	11	42.3%	
Mode of trauma	RTA	10	71.4%	24	92.3%	0.159
	Fall	4	28.6%	2	7.7%	
Fracture type	Schatzker II	9	64.3%	13	50.0%	0.383
	Schatzker III	3	21.4%	8	30.8%	
	Schatzker IV	1	7.1%	5	19.2%	
	Schatzker V	1	7.1%	0	0.0%	
Use of bone graft	Yes	4	28.6%	20	76.9%	0.003
	No	10	71.4%	6	23.1%	
Preoperative displacement and fragmentation	Yes (complex fracture)	10	71.4%	3	11.5%	< 0.001
	No (simple fracture)	4	28.6%	23	88.5%	
Special habits	Smoking	7	50.0%	3	11.5%	0.018
	non	7	50.0%	23	88.5%	
Weight-bearing time	>10 w	6	42.9%	24	92.3%	< 0.001
	6-8 w Partial	3	21.4%	2	7.7%	
	6-8 w full	5	35.7%	0	0.0%	

Age

**Figure 1:**
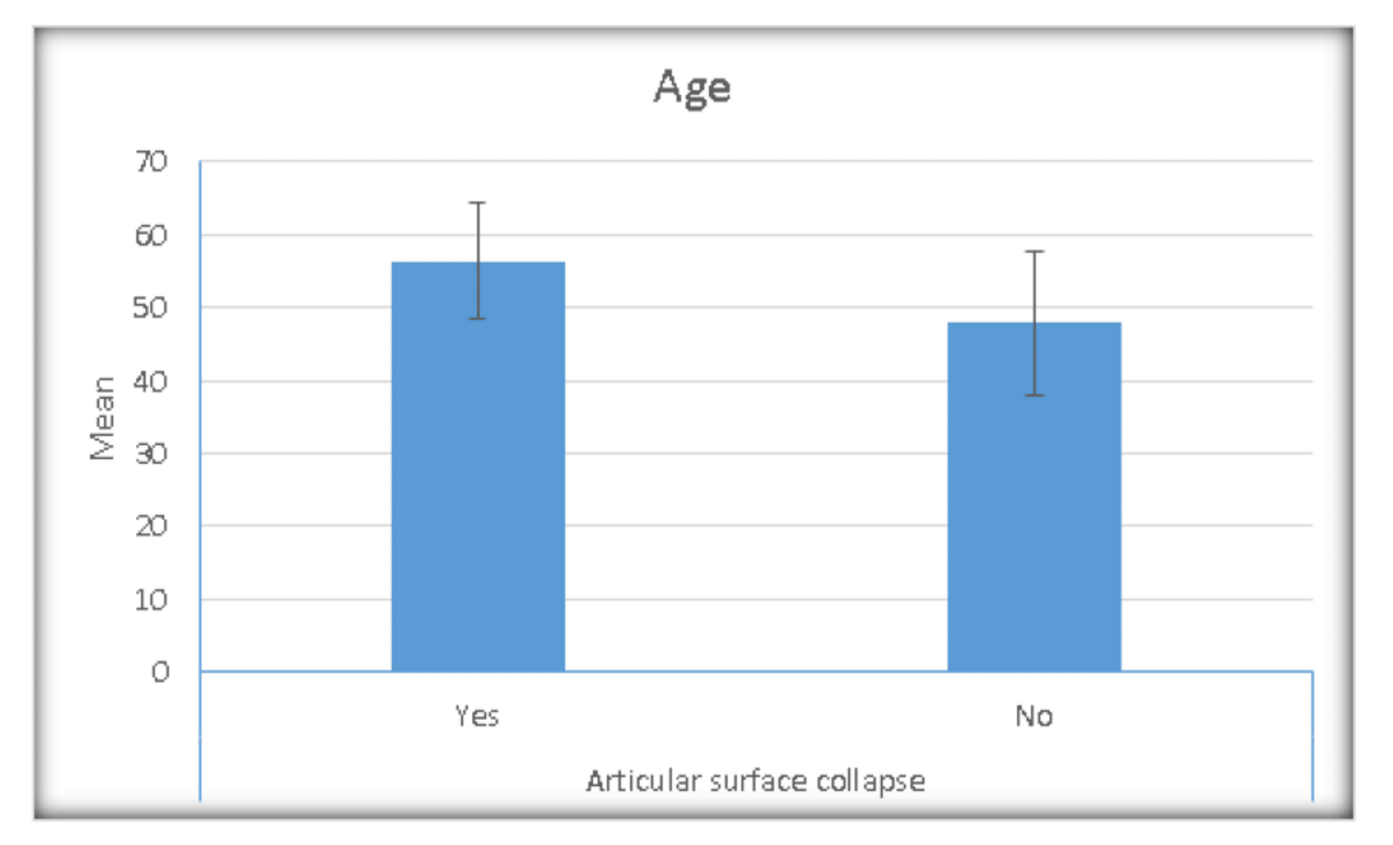
Bar chart showing the relationship between patient age and the occurrence of articular surface collapse

Figure 1 shows the relationship between patient age and the occurrence of articular surface collapse. The mean age of patients with postoperative collapsed articular surface was 56.6 years, showing a significant P value of 0.009. Statistical analysis revealed a significant association between patient age and the development of postoperative articular surface collapse. Patients who experienced articular surface collapse demonstrated a notably higher mean age of 56.3 years (SD ± 7.97 years) compared to those who maintained intact articular surfaces, whose mean age was 47.88 years (SD ± 9.93 years). This age difference proved to be statistically significant with a p-value of 0.009, indicating that advancing age represents a significant risk factor for the development of postoperative articular surface depression. The approximately 8.4-year age difference between the two groups suggests that older patients may be at increased risk for loss of reduction, potentially due to factors such as decreased bone quality, reduced healing capacity, or increased susceptibility to osteoporotic changes that compromise the structural integrity of the tibial plateau. 

Total function score in relation to articular surface collapse

A significant P value indicates that articular surface depression affected the patients' walking ability and pain, which in turn affected their overall function score (Table 5). There were significant associations with increased pain (100% of collapse patients reported pain vs. 23.1% without collapse, p<0.001), reduced walking capacity (7.1% maintained normal walking vs. 76.9% in the non-collapse group, p<0.001), and poorer total function scores (0% excellent results vs. 80.8% in controls, p<0.001) at the six-month follow-up.

**Table 5:** Association between function score and postoperative articular surface collapse Analyzed using chi-square tests with significance at p<0.05

Parameters		Articular surface collapse				
		Yes		No		P value
		Frequency	Percentage	Frequency	Percentage	
Pain	Constant pain after activity	4	28.6%	0	0.0%	< 0.001
	Stabbing pain in certain positions	6	42.9%	0	0.0%	
	Occasional	4	28.6%	7	26.9%	
	No pain	0	0.0%	19	73.1%	
Walking capacity	Walking indoors only	2	14.3%	0	0.0%	< 0.001
	Walking outdoors (<15m)	1	7.1%	0	0.0%	
	Walking outdoors (15m-1h)	10	71.4%	5	19.2%	
	Walking outdoors (>1 h)	1	7.1%	1	3.8%	
	Normal	0	0.0%	20	76.9%	
Knee extension	Normal	12	85.7%	25	96.2%	0.276
	Lack of extension (<10)	2	14.3%	1	3.8%	
Range of motion	At least 120	6	42.9%	4	15.4%	0.123
	Full	8	57.1%	22	84.6%	
Stability	Normal	11	78.6%	24	92.3%	0.322
	Abnormal stability in 20 degrees flexion	3	21.4%	2	7.7%	
Quadriceps Power	4	3	21.4%	2	7.7%	0.322
	5	11	78.6%	24	92.3%	
Total functional score at 6 months	Poor	3	21.4%	0	0.0%	< 0.001
	Fair	4	28.6%	2	7.7%	
	Good	7	50.0%	3	11.5%	
	Excellent	0	0.0%	21	80.8%	

Total radiological score in relation to articular surface collapse

The collapse of the articular surface significantly influences radiographic score findings compared to other patients, as indicated by a notable P value (Table 6). There were significant associations between articular surface collapse and articular step-off (100% of collapse cases showed >5mm displacement vs. 0% in controls, p<0.001), angulation (92.9% of collapse cases had <10° angulation vs. 88.5% in controls, p=0.015), and poorer total radiological scores (0% excellent results vs. 85.5% in controls, p<0.001).

**Table 6:** : Association of Radiological score with postoperative articular surface collapse Analyzed using chi-square or Fisher's exact tests with significance at p<0.05

Parameters		Articular surface collapse				
		Yes		No		P value
		Frequency	Percentage	Frequency	Percentage	
Articular step off	No	0	0.0%	26	100.0%	< 0.001
	<5 mm	8	57.1%	0	0.0%	
	6-10 mm	6	42.9%	0	0.0%	
Widening	No	14	100.0%	26	100.0%	-----
Angulation	<10	13	92.9%	3	11.5%	< 0.001
	No	1	7.1%	23	88.5%	
Osteoarthritis	No	0	0.0%	15	57.7%	< 0.001
	Progression by 1 grade	10	71.4%	11	42.3%	
	Progression by >1 grade	4	28.6%	0	0.0%	
Total radiological score at 6 months	Fair	8	57.1%	0	0.0%	< 0.001
	Good	6	42.9%	3	11.5%	
	Excellent	0	0.0%	23	88.5%	

Multivariate regression

Multivariate logistic regression identified three independent predictors of articular collapse: absence of bone grafting (OR=0.067, p=0.039), preoperative fracture complexity (OR=49.576, p=0.005), and early full weight-bearing (<10 weeks, OR=25.046, p=0.015) (Table 7).

**Table 7:** Multivariate logistic regression

Parameters		P value	OR	95% C.I.	
				Lower	Upper
Articular surface collapse	Use of bone graft	0.039	0.067	0.005	0.873
	Preoperative displacement and fragmentation	0.005	49.576	3.175	774.024
	Full weight-bearing time < 10 W	0.015	25.046	1.861	336.993

Imaging findings from two patients from the study

Patient A

**Figure 2:**
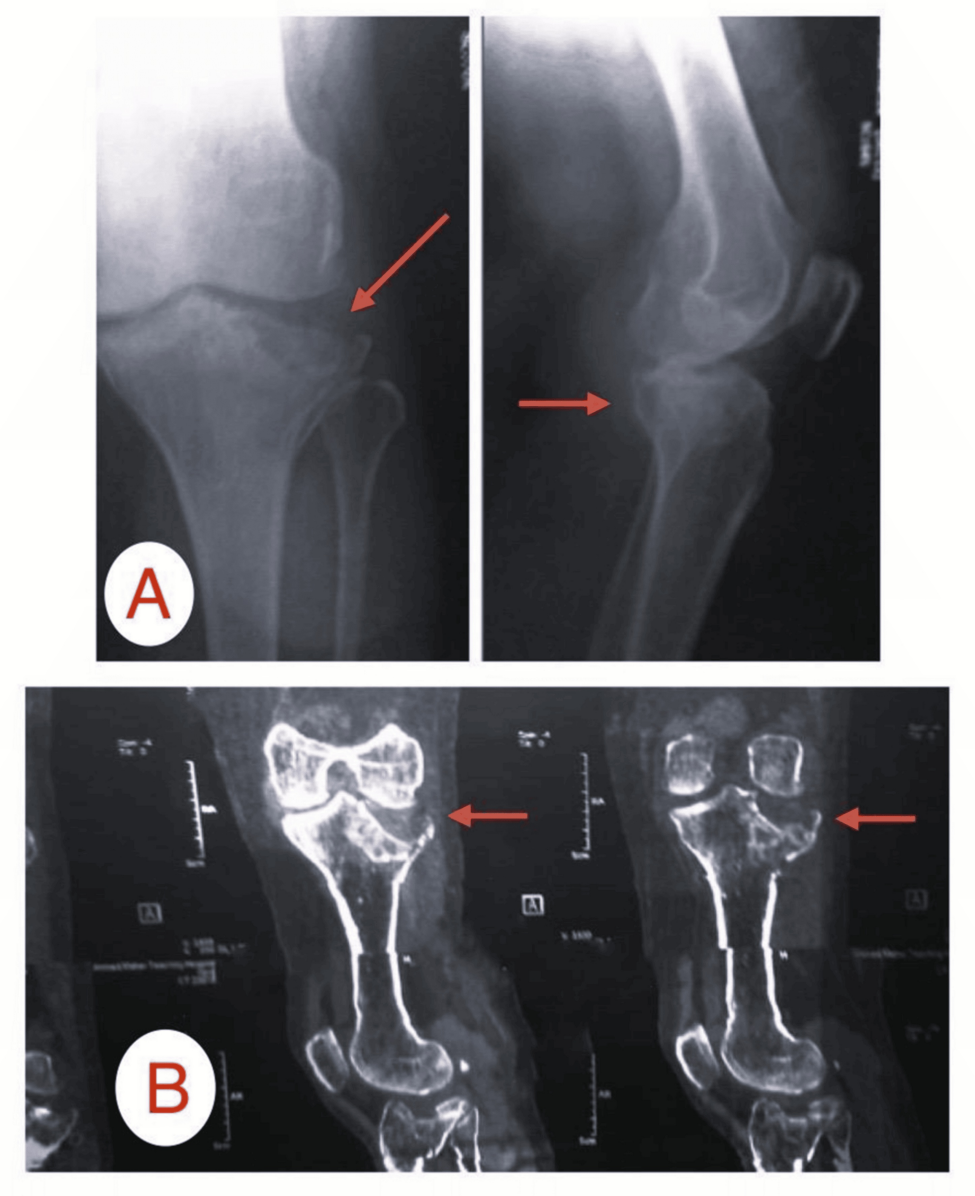
Preoperative images of Patient A: (A) X-ray showing a depressed fracture of the tibial plateau, (B) CT scan showing a depressed lateral tibial plateau fracture

**Figure 3:**
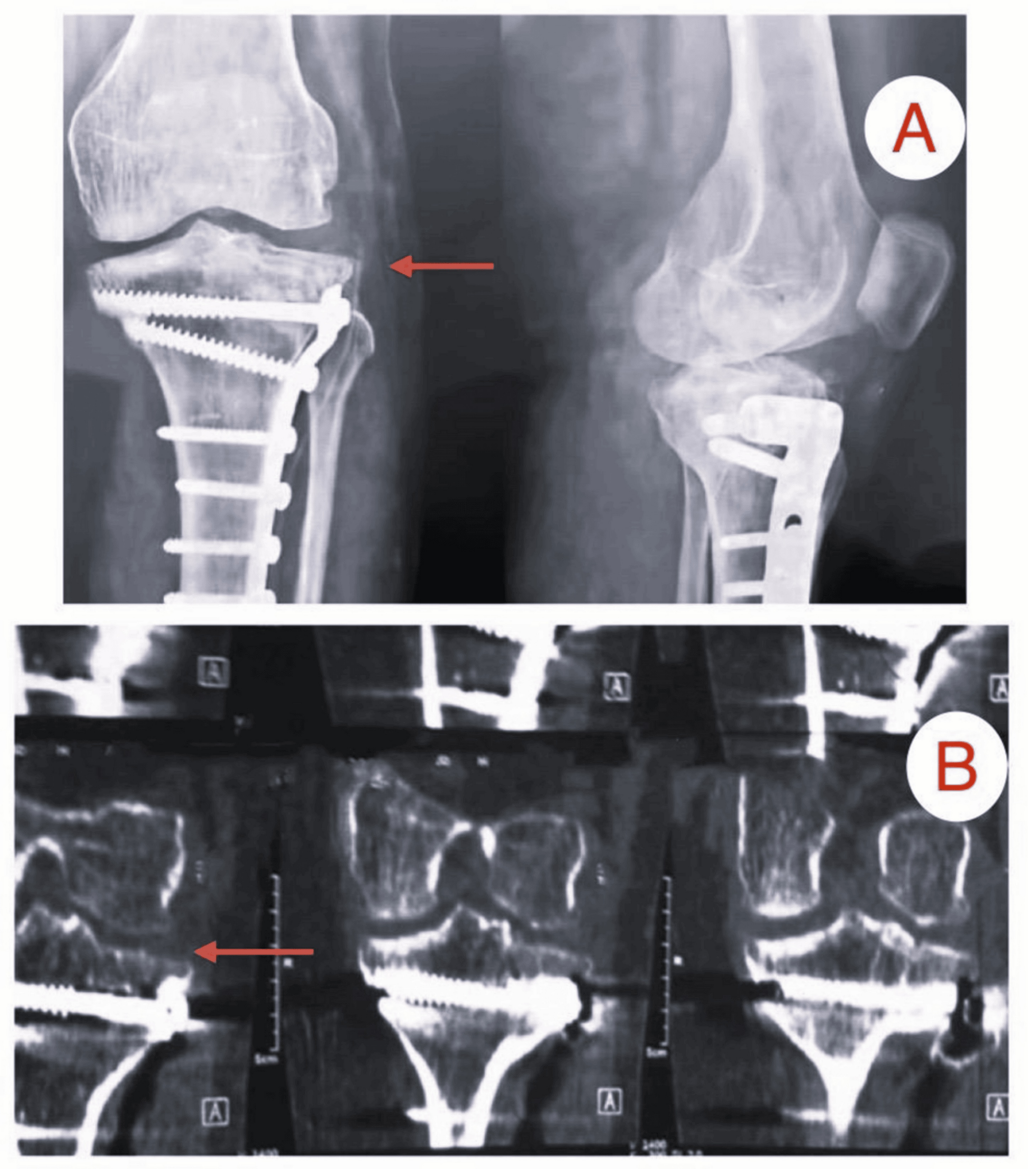
Immediate postoperative images of Patient A: (A) X-ray showing reduction of depressed plateau fracture, (B) CT scan showing an elevated reduced plateau fracture

**Figure 4:**
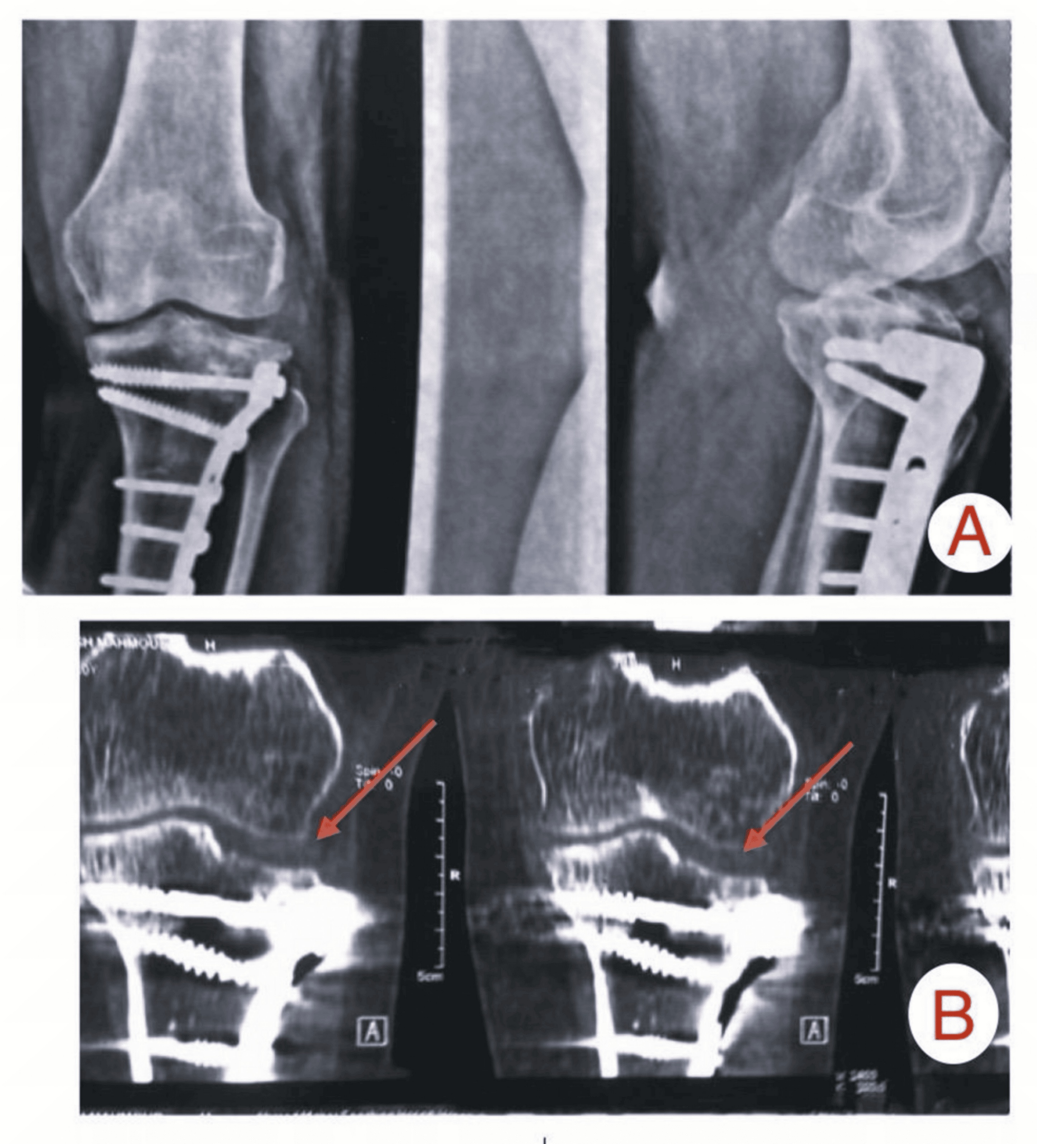
Six-month follow-up (A) X-ray and (B) CT scan images of Patient A showing secondary depression of the reduced lateral tibial plateau articular surface

This was a female patient, 48 years old, with a fracture of the tibial plateau (Schatzker type III) resulting from an accident involving a fall (Figures 2-4).

Patient B

**Figure 5:**
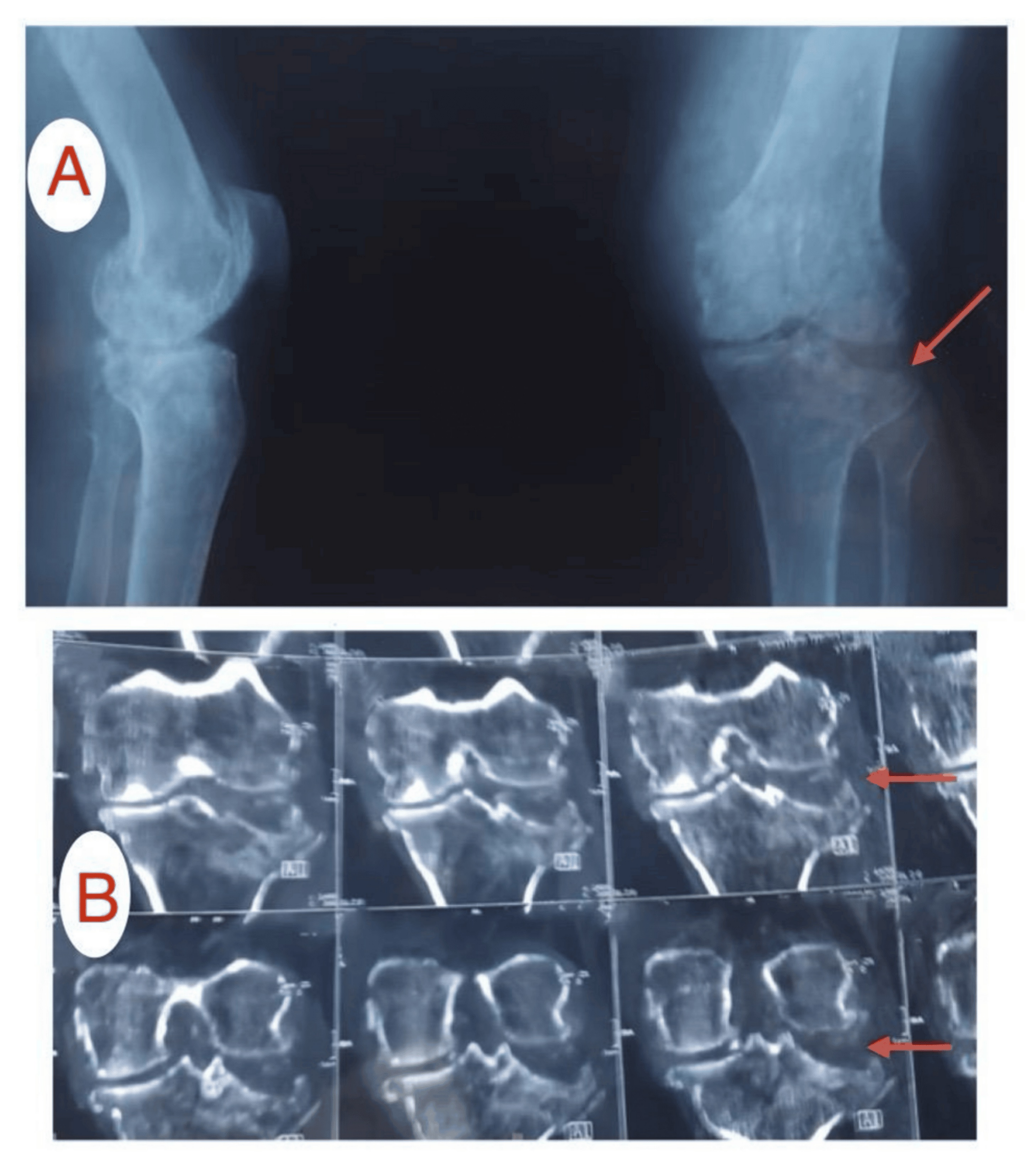
Preoperative images of patient B: (A) X-ray showing a split depressed fracture of the lateral tibial plateau, and (B) CT scan showing a depression fracture in the lateral tibial plateau

**Figure 6:**
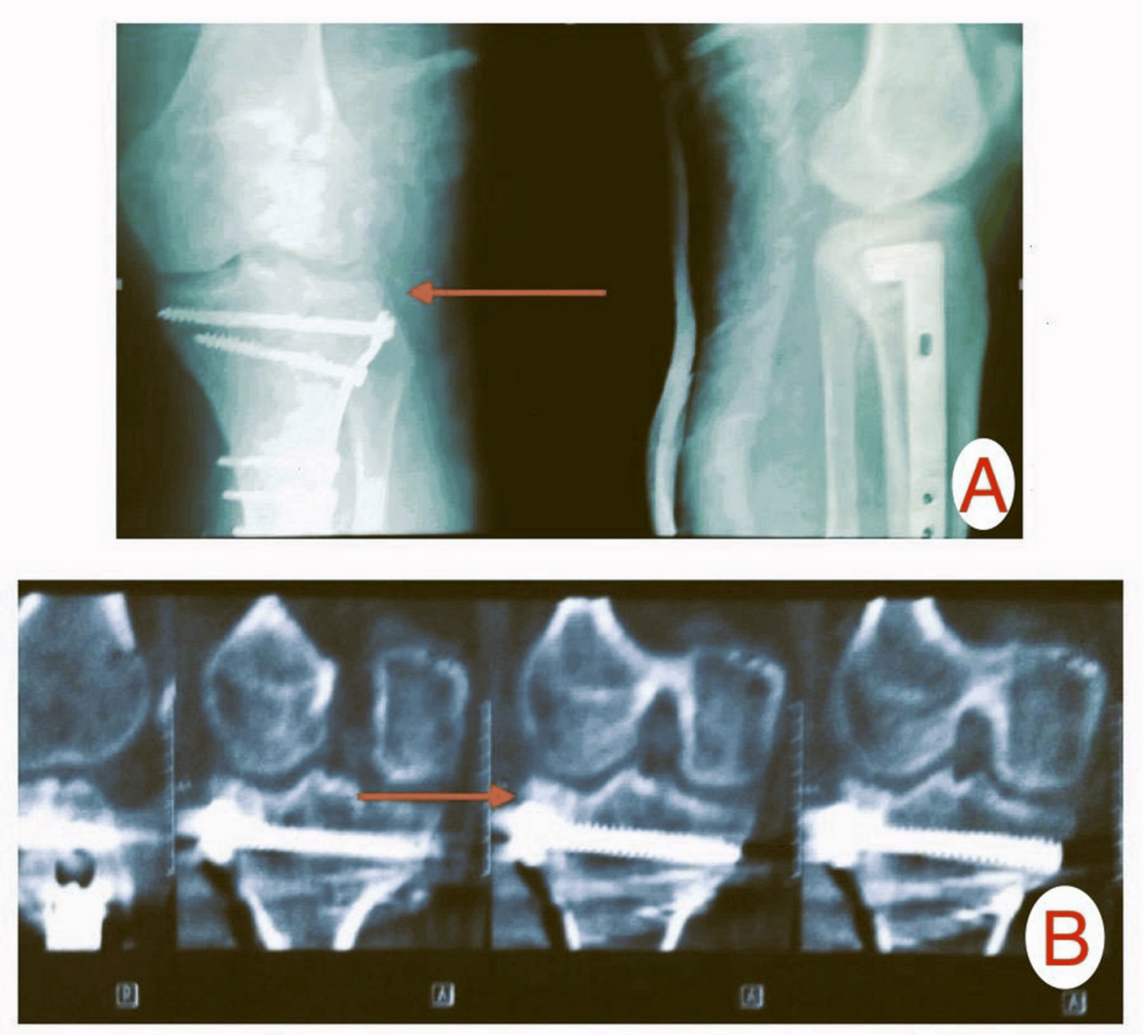
Immediate postoperative images of Patient B: (A) X-ray showing a reduced tibial plateau fracture, (B) CT scan showing reduction and elevation of the depressed fracture and restoration of articular congruity

**Figure 7:**
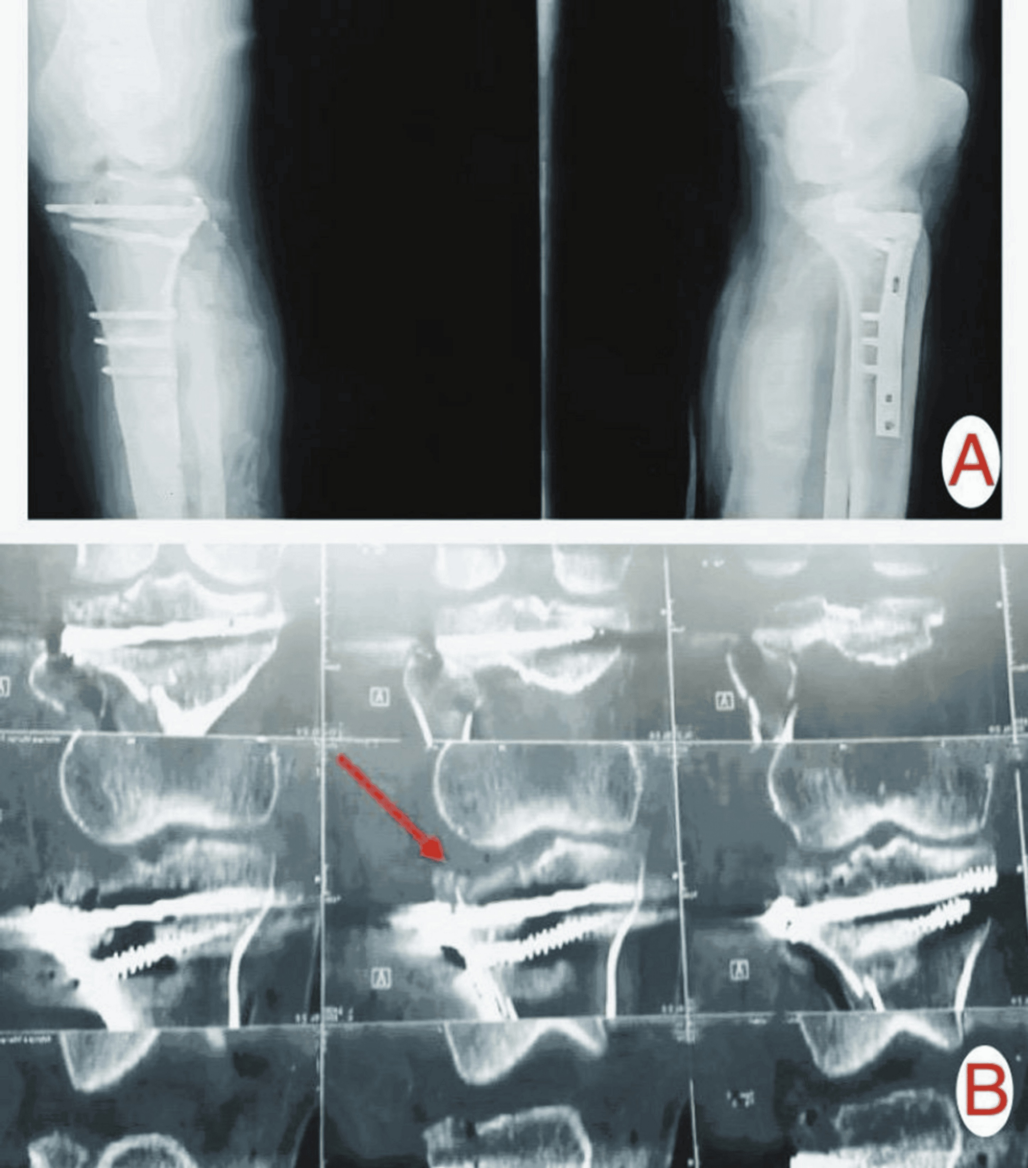
Six-month follow-up (A) X-ray, and (B) CT scan images of Patient B showing secondary depression of the reduced lateral tibial plateau articular surface

This was a male patient, 55 years old, with fracture of the tibial plateau (Schatzker type II), from a road traffic accident (Figures 5-7).

## Discussion

TBFs present significant challenges, especially when joint depression is accompanied by metaphyseal comminution. The primary objective in these fractures is to attain, if feasible, an anatomic reduction of the articular surface, thereby restoring joint congruity and mechanical alignment to enhance functional outcomes and decrease the incidence of post-traumatic arthritis. Fractures with significant depressed articular surfaces necessitate elevating the depressed fragments and stable internal fixation [18].

The prevalence and extent of secondary articular surface collapse were examined across 17 studies involving 553 cases. These studies exhibited notable methodological variability. A collapse of the joint surface greater than 2 mm has been proposed as a clinically significant threshold by several researchers [19].

Schatzker and co-authors initially defined articular depression as a measurement exceeding 4 mm on plain radiographs [20]. Additionally, various studies have highlighted a positive correlation between the severity of fractures, the resulting tibial plateau incongruence, and the development of post-traumatic osteoarthritis. Brown et al. demonstrated that a step-off greater than 1.5 mm leads to considerable contact stresses on the articular surface [21], while research using animal models suggests that osteoarthritis develops when a step-off surpasses 2-5 mm [22]. According to Parkkinen et al., significant post-traumatic arthritis in patients with TPF was predicted by postoperative articular depression of more than 2 mm [23].

In our study, a threshold of articular incongruence greater than 2 mm was adopted to define inadequate joint step-off, which was applied to both the initial reduction and any subsequent collapse. The threshold of ≥2 mm for secondary articular collapse was used as the cutoff point for evaluating postoperative outcomes.

In the review by Hartwich et al., secondary collapse consistently served as a key metric for assessing the mechanical environment's effectiveness [18]. However, the methods for evaluating collapse and the timing of its detection varied significantly across the reviewed studies; many utilized plain radiographs and CT scans for initial fracture evaluation and preoperative assessment, but few studies employed CT during follow-up. The sensitivity of CT scans in measuring step-off and the surface area of depression is not consistently addressed in the literature, and further studies should explore the relationship between radiological findings and clinical outcomes.

This study aimed to evaluate postoperative articular congruity and to quantify the articular surface collapse following open reduction and restoration of depressed fractures in 40 patients. We analyzed the data in relation to factors such as patient age, sex distribution, injury mechanism, fracture type, and lifestyle habits. The second objective was to assess surgical and patient-related factors contributing to the observed outcomes. Data were analyzed for statistically significant correlations between these variables and clinical and radiographic outcomes.

TPF is frequently observed in individuals of active, productive age (31-50) due to high-energy trauma, as well as in the elderly, who may be more prone to fractures due to decreased bone density. The average age of the patients in this study was 50.85 ± 10.05 years, with males comprising 52.5% of the cohort. Road traffic accidents (RTAs) accounted for 85% of the injuries.

Fourteen (35%) patients showed postoperative articular surface collapse during the follow-up evaluation. The average age of this cohort was 56.6 years (SD 7.97), and 57.1% were female; of the 19 female patients in the study, eight experienced collapse, compared to only six out of 21 males.

We identified a significant correlation between age and secondary articular depression, with an average age of 56.6 years in the group experiencing collapse and a p-value of 0.009. This finding aligns with the results of Ali et al., who found that 85% of older patients experienced loss of reduction [24]. Similarly, Honkonen et al. observed that achieving stable fixation was particularly challenging in older patients, with a high risk of reduction loss despite internal fixation and bone grafting [25].

The degree of fragmentation and preoperative displacement was associated with loose reduction, primarily due to the high-energy forces involved in the injury and/or the presence of osteoporosis. These findings are consistent with the research of Kennedy and Bailey, who demonstrated that both the intensity of the injury forces and the extent of osteopenia significantly contribute to the fragmentation and subsequent displacement of the fracture fragments [26]. Fractures exhibiting greater fragmentation and significant articular impaction, along with loss of metaphyseal cancellous support, are more prone to postoperative disintegration [24]. Our findings revealed that complex fractures were present in 10 out of 14 patients with postoperative depression (p-value > 0.001).

It has been clear through the study that fractures associated with depression exceeding 4 mm, separations greater than 2 mm, and/or comminution, varus, or valgus instability (complex fractures) had a greater chance for collapse, particularly in the elderly or early weight-bearing, even with the use of bone grafting intervention. Eight out of 10 patients who had complex fractures resulting in subsequent collapse were above 50 years of age; however, the other two patients were 49 and 46 years of age. Moreover, six of these patients were female. 

Through the study, the effect of smoking on the results was observed with a P value of 0.018. Furthermore, it was clearly seen that using a bone graft made a difference, as 10 out of 14 patients in the group and 16 patients in the entire study who didn't have a graft experienced joint collapse with a p-value of 0.003.

This corresponds with Huang et al.'s findings: the entire 25 TPF patients had been handled during the index procedures without bone grafting [11]. All patients exhibited significant bone deficits ranging between 69 cm³ and 190 cm³ after the revision operations. Lachiewicz and Funcik examined the elements affecting the ORIF outcomes of 43 tibial plateau fractures in 1990, and eight patients (19%) with no bone graft used had the worst outcomes [27]. Except in situations of the simple type I split or in young patients with high bone quality and minor comminution, bone grafting should be used to raise the depressed fragment and fix the varus or valgus deformity.

Instances of noncompliance with weight-bearing instructions were observed in our study in both young and elderly patients. A significant association exists between premature weight bearing and loss of reduction. The analysis of younger patients exhibiting loss of reduction reveals a significant correlation with premature weight bearing. Ten patients exhibited early weight-bearing. In follow-up, 60% of patients who initiated early partial weight-bearing exhibited articular depression, whereas 100% of those who resumed early full weight-bearing experienced collapse (p-value < 0.001). Only six patients out of 30 who adhered to the instructions and began weight bearing during the 10th postoperative week encountered collapse.

This was consistent with research by Huang et al. that found a significant correlation (p < 0.05) between loss of reduction and early weight bearing [11]. Two patients in their study who disregarded guidance and discarded their walking aids experienced a loss of reduction during the first six weeks. During the first six weeks following surgery, eight more patients began premature partial weight bearing; of these, six experienced a drawback (75%), and nine patients (25%) experienced reduction losses out of the 36 who refrained from full weight bearing until 10 weeks later.

A few studies point to noncompliance with mobilization protocol as a contributing factor to plateau collapse or varus malunion. Moore et al. reported a significant average loss of fracture reduction of 2.7 mm attributed to noncompliance among patients who failed to adhere to non-weight-bearing recommendations post-discharge [28]. In the study by Raikin and Froimson, noncompliant patients experienced a 30% rate of loss reduction and varus malunion, even with the use of a strong external fixator [29].

The total function scores of the 14 patients with postoperative articular surface depression ranged from good to fair to poor, with no excellent scores. Among the remaining 26 patients (without articular surface depression), 21 exhibited excellent results, with a significant P value of > 0.001, suggesting a robust correlation between postoperative collapse and functional outcomes. Data showed a 100% association for postoperative collapse and pain; four patients experienced constant pain after activity, six patients had stabbing pain in certain positions, and occasional pain was noted in the remaining four patients (p-value > 0.001). Another significant effect on the function score, with a p-value of >0.001, is that all patients with articular surface collapse experienced a decrease in their walking ability.

The radiographic score was significantly influenced by the secondary collapse of the articular surface, with a P value of less than 0.001. Among the 14 patients with postoperative articular surface depression, articular step-off and progression of osteoarthritis by grade 1 or more than grade 1 were observed. Notably, 13 of the 14 patients exhibited angulation of less than 10 degrees.

Directions for future research

The substantial appositional cancellous bone along the cross-sectional area and robust vascularization of the proximal tibia render a nonunion TBF unlikely. A high rate of loss of articular surface reduction in our study (35%) raises questions for further research: (i) What are the optimal fixation techniques and implant designs for ensuring a stable reduction of osteoporotic bone? (ii) What are the comparative long-term clinical outcomes for patients who fail to maintain the anatomical reconstruction target on which the operation decision was dependent? The responses to the above questions will certainly impact our further management of TPFs.

Limitations of the study

One limitation of the study is that we did not include BMI, which is believed to considerably influence joint surface depression, and the analysis of its effect on articular surface depression. We advocate for the incorporation of this variable in further research and the comparison of the effects of autologous bone grafting with graft replacements to achieve superior outcomes.

## Conclusions

It makes sense to measure the loss of joint surface reduction using the same imaging criteria that indicate when to perform ORIF. Utilizing these criteria, the frequency of secondary articular depression was found to be high and is particularly prevalent in older people. There is consensus regarding the necessity of surgical anatomical restoration of the articular surface in younger patients, a goal that is typically unattainable by nonoperative treatments. In the older demographic, the incidence of articular surface reduction loss was significantly high, necessitating a careful evaluation of the advantages of restoring proximal tibial morphology against the possible risks of collapse and surgical sequelae. In geriatric individuals, it may be advantageous to have a limited objective of achieving alignment and stability.

Furthermore, surgery may offer no benefit to patients who cannot adhere to instructions concerning protected weight bearing. However, by employing a retrospective chart review of interval attendances, the authors of our study acknowledge the potential of inaccurately timing the precise onset of weight bearing. According to our findings, refraining from weight-bearing for at least 10 weeks is advised. Bone graft augmentation remains the gold standard for addressing metaphyseal defects in load-bearing bones like the tibial plateau. The degree of fracture complexity plays a significant role in postoperative depression, especially when reduction is followed by inadequate grafting and/or premature weight-bearing. In our study, the use of bone grafts and smoking cessation were significantly associated with a lower incidence of articular collapse and improved clinical and radiological outcomes. These findings suggest that these modifiable factors play a protective role in the management of tibial plateau fractures.
